# Interim Guidance for Prevention and Treatment of Monkeypox in Persons with HIV Infection — United States, August 2022

**DOI:** 10.15585/mmwr.mm7132e4

**Published:** 2022-08-12

**Authors:** Jesse O’Shea, Thomas D. Filardo, Sapna Bamrah Morris, John Weiser, Brett Petersen, John T. Brooks

**Affiliations:** ^1^CDC Monkeypox Response; ^2^Epidemic Intelligence Service, CDC.

*Monkeypox virus*, an orthopoxvirus sharing clinical features with smallpox virus, is endemic in several countries in Central and West Africa. The last reported outbreak in the United States, in 2003, was linked to contact with infected prairie dogs that had been housed or transported with African rodents imported from Ghana (*1*). Since May 2022, the World Health Organization (WHO) has reported a multinational outbreak of monkeypox centered in Europe and North America, with approximately 25,000 cases reported worldwide; the current outbreak is disproportionately affecting gay, bisexual, and other men who have sex with men (MSM) (*2*). Monkeypox was declared a public health emergency in the United States on August 4, 2022.^†^ Available summary surveillance data from the European Union, England, and the United States indicate that among MSM patients with monkeypox for whom HIV status is known, 28%–51% have HIV infection (*3*–*10*). Treatment of monkeypox with tecovirimat as a first-line agent is available through CDC for compassionate use through an investigational drug protocol. No identified drug interactions would preclude coadministration of tecovirimat with antiretroviral therapy (ART) for HIV infection. Pre- and postexposure prophylaxis can be considered with JYNNEOS vaccine, if indicated. Although data are limited for monkeypox in patients with HIV, prompt diagnosis, treatment, and prevention might reduce the risk for adverse outcomes and limit monkeypox spread. Prevention and treatment considerations will be updated as more information becomes available.

## Background

**Signs and Symptoms:** Classically, monkeypox occurs in three stages. After an incubation period of approximately 1–2 weeks, a prodrome, characterized by fever and lymphadenopathy occurs, which is followed by the onset of a deep-seated vesicular or pustular rash that often begins centrally and spreads to the limbs (*11*). Transmission of monkeypox can occur through direct contact with the infectious rash, scabs, or body fluids, through respiratory secretions during prolonged face-to-face contact or intimate physical contact, or through touching items, such as clothing or linens, that previously touched a patient’s infectious rash or body fluids.^§^ Patients are considered contagious until the scabs have crusted over and fallen off and a fresh layer of intact skin has formed underneath.

Reports from the current outbreak suggest transmission patterns and clinical manifestations might not follow the classic presentation of monkeypox (*5*–*10*). Although any person can acquire monkeypox, epidemiologic data indicate that transmission is currently most intense among interconnected networks of sexually active MSM, with transmission occurring primarily through intimate skin-to-skin contact during sex (*6*). Prodrome or systemic symptoms do not always occur or precede the rash. Mucosal involvement occurs in approximately 40% of cases, including genital, perianal, and oropharyngeal lesions (*5*). Genital and perianal lesions can be associated with severe and painful proctitis, urethritis, phimosis, and balanitis. Oropharyngeal symptoms, including symptoms resulting from tonsillitis and epiglottitis, can be associated with pain or difficulty swallowing.

**Treatment:** There are no Food and Drug Administration (FDA)–approved treatments for monkeypox. However, drugs that are approved for treatment of smallpox and cytomegalovirus might have activity against *Monkeypox virus*. Tecovirimat is an antiviral medication available in oral and intravenous formulations. Animal studies have shown that tecovirimat is effective in treating orthopoxvirus-induced disease (*12*). Data are not available on the effectiveness of tecovirimat in treating monkeypox in humans; however, a case report from the United Kingdom suggested that tecovirimat might shorten the duration of illness and of viral shedding (*13*). Human clinical trials indicate that the drug is safe and tolerable with only minor side effects (*14*). Randomized controlled trials in humans are underway to further assess safety as well as efficacy in treating monkeypox. Tecovirimat is available from the Strategic National Stockpile (SNS) and is administered under an expanded access (i.e., compassionate use) Investigational New Drug (EA-IND) protocol held by CDC.^¶^

Other treatments that can be considered in severe cases include vaccinia immune globulin intravenous (VIGIV), cidofovir, and brincidofovir. Cidofovir and brincidofovir have proven activity against poxviruses in in vitro and animal studies, but only cidofovir is currently available either commercially or from the SNS. VIGIV is available from the SNS and is administered under an EA-IND protocol for monkeypox. At this time, it is unknown whether a person with severe monkeypox will benefit from treatment with VIGIV, cidofovir, or brincidofovir because effectiveness data are not available.

**Pre- and Postexposure Prophylaxis**: The only form of pre-exposure prophylaxis available or authorized for monkeypox is vaccination, which currently is recommended for persons at risk for occupational exposure to orthopoxviruses, such as laboratory personnel performing diagnostic testing for *Monkeypox virus* and members of health care worker response teams designated by appropriate public health and antiterror authorities (*15*). Routine immunization of all health care workers against smallpox or monkeypox is not currently recommended.**

Postexposure prophylaxis can be considered after exposure to monkeypox.^††^ Although the use of smallpox vaccines for postexposure prophylaxis has not been studied in the context of monkeypox outbreaks, early administration of vaccines (≤4 days after exposure) might prevent monkeypox, and later use (5–14 days after exposure) might decrease the severity of monkeypox if infection occurs (*16*,*17*). Vaccination given after the onset of signs or symptoms of monkeypox is not expected to provide benefit.^§§^

Two vaccines are licensed by FDA for the prevention of orthopoxvirus infections. JYNNEOS is a live virus vaccine that uses nonreplicating modified vaccinia Ankara (MVA) which is licensed for prevention of smallpox and monkeypox in adults aged ≥18 years (*18*). Because JYNNEOS contains replication-deficient MVA, it does not present a risk for disseminated infection, autoinoculation, or transmission to others (*15*). JYNNEOS vaccine is administered as a series of two doses given 28 days apart (*18*). ACAM2000 is a replication-competent live vaccinia virus vaccine licensed for prevention of smallpox that is administered as a single dose (*19*). ACAM2000 was derived from Dryvax, the vaccine used in the eradication of smallpox (*19*).

## Monkeypox in Persons with HIV Infection

**Clinical Presentation and Outcomes:** It is currently not known whether HIV infection affects a person’s risk for acquiring monkeypox. MSM with HIV infection are at present disproportionately represented among monkeypox cases. However, ascertaining the relative roles that exposure and biologic risks play in this disproportionality is challenging. Sexual behavior that confers risk for HIV acquisition also increases risk for acquiring other sexually transmitted infections (STIs) leading to a similar disproportionate overrepresentation of MSM with HIV among STI cases (*20*); risk for monkeypox through sexual contact is likely similarly increased. Although it is possible that poorly controlled HIV would increase risk for monkeypox after exposure, evidence from other diseases suggests that persons with HIV infection who are receiving ART and have robust CD4 counts are not at increased risk for most infections, including opportunistic infections, and therefore might not be at increased risk for monkeypox after exposure.^¶¶^

Available data indicate that persons with advanced and uncontrolled HIV infection might be at higher risk for severe or prolonged monkeypox disease following infection. In a 2017–2018 case series describing 122 Nigerian patients with monkeypox caused by the same strain responsible for the current outbreak, four of the seven deaths occurred among persons with untreated advanced HIV infection; however, information about the overall proportion of patients with HIV infection was not available, precluding the ability to determine whether this mortality was disproportionately large (*21*). A second 2017–2018 series of 40 monkeypox cases, also from Nigeria, included nine persons with HIV infection for whom clinical data relevant to HIV status were provided; CD4 cell counts ranged from 20 to 357 per *μL*, and most patients had either failed ART or had newly diagnosed HIV infection, suggesting a lack of viral suppression. Two of nine patients with HIV in that case series died. Compared with other patients with monkeypox, those with HIV infection had higher rates of secondary bacterial infection, more prolonged illness (and thereby also longer period of infectiousness), as well as a higher likelihood of having a confluent or partially confluent rash rather than discrete lesions (*22*). In contrast, recent reports from European countries where most patients are receiving effective ART have noted no deaths or evident excess in hospitalizations among persons with HIV infection and monkeypox to date (*3*,*4*,*6*). In addition, WHO has stated that a more severe disease course has not been reported in persons with HIV infection who are receiving ART and have a robust immune system (*23*), a finding supported by recent large cohort studies (*5*,*7*,*8*).

**Management of patients with HIV infection and monkeypox:** ART and opportunistic infection prophylaxis should be continued in all persons with HIV infection who acquire monkeypox (Table 1). Treatment interruption might lead to rebound HIV viremia that could complicate the management of monkeypox, including worsening illness severity.*** Persons receiving ART for HIV pre-exposure prophylaxis or postexposure prophylaxis should likewise continue taking these medications. Persons with newly diagnosed HIV infection at the time of monkeypox diagnosis should commence ART as soon as possible, in consultation with an expert in HIV care, if needed. Monkeypox diagnosis has been reported concurrent with diagnosis of acute HIV infection and other STIs, highlighting the importance of testing for these infections when monkeypox is suspected or diagnosed (*24*).

**TABLE 1:** Recommendations for management of persons with HIV infection and monkeypox — United States, August 2022

Patient group and treatment	Recommendations/Precautions	Availability/Effectiveness in treating monkeypox
**HIV management for persons with monkeypox**		
Known HIV infection	Continue ART and opportunistic infection prophylaxis as indicated	NA
Newly diagnosed HIV	Begin ART as soon as possible	NA
HIV pre-exposure prophylaxis	Continue treatment or start, as indicated	NA
HIV postexposure prophylaxis	Continue treatment or start, as indicated	NA
Monkeypox management for persons with HIV*		
Tecovirimat (TPOXX, ST-246)	Review potential interactions with ART	Available from SNS
		Oral and intravenous formulations available
Cidofovir (Vistide)	Contraindicated if serum creatinine >1.5 mg/dL	Available from SNS
		Effectiveness in treating monkeypox unknown
Brincidofovir (CMX001, Tembexa)	Might cause increases in serum transaminases and bilirubin	Not available from SNS
		Effectiveness in treating monkeypox unknown
Vaccinia immune globulin intravenous	Might be considered in severe cases	Available from SNS
		Effectiveness in treating monkeypox unknown
**Monkeypox pre-exposure prophylaxis^†^**		
JYNNEOS^§^ vaccine (2-dose, nonreplicating live vaccinia virus vaccine)	Safety and immunogenicity similar in persons with and without HIV infection	Licensed for prevention of orthopoxvirus infections, including monkeypox^¶^
**Monkeypox postexposure prophylaxis^†^**		
JYNNEOS^§^ vaccine (2-dose, nonreplicating live vaccinia virus vaccine)	Safety and immunogenicity similar in persons with and without HIV infection	Limited available data. If administered ≤4 days after exposure, might prevent infection; administration ≥5 days after exposure might decrease severity of disease if infection occurs.

**Abbreviations**: ART = antiretroviral therapy; FDA = Food and Drug Administration; NA = not applicable; SNS = Strategic National Stockpile.

* https://www.cdc.gov/poxvirus/monkeypox/clinicians/treatment.html

^†^ ACAM2000 is a replication-competent vaccina virus vaccine that is licensed for prevention of smallpox. ACAM2000 should not be used in persons with HIV infection, regardless of immune status. https://www.fda.gov/media/75792/download

^§^ https://www.fda.gov/media/131078/download

^¶^ https://www.cdc.gov/poxvirus/monkeypox/considerations-for-monkeypox-vaccination.html

Treatment of monkeypox should be considered among persons with HIV infection, taking into account disease severity, degree of immunosuppression, or vulnerable sites of infection (e.g., the genitals or anus).^†††^ Tecovirimat is the first-line medication recommended for treatment of monkeypox, including among persons with HIV infection. Clinically relevant interactions among tecovirimat, cidofovir, and brincidofovir and certain ARTs are known and should be considered when selecting treatment (Table 2). However, none of the identified drug interactions should preclude coadministration of tecovirimat and antiretroviral therapy. Cidofovir is contraindicated in patients with serum creatinine >1.5 mg/dL because of the associated nephrotoxicity. There are no specific contraindications for use of VIGIV among persons with HIV infection.

**TABLE 2:** Treatments for monkeypox and clinically relevant drug interactions with antiretroviral therapies

Monkeypox treatment	ART	Mechanism	Clinical comments
Tecovirimat	Doravirine (DOR)	Induction of CYP3A4	Consultation with local pharmacists is suggested. Interaction may result in a reduction in NNRTI and MVC levels. Per Liverpool HIV interactions database, dose increases could be considered for these antiretroviral medications during therapy and for 2 wks after completion of tecovirimat therapy.* However, based on evidence graded very low quality and the short treatment course of tecovirimat, some experts believe neither dose adjustments nor additional ART are needed.^†^
	Rilpivirine (RPV)		
	Maraviroc (MVC)		
	Long-acting cabotegravir/RPV	Induction of CYP3A4	Consultation with local pharmacists is suggested. Interaction might result in a reduction in RPV levels. Per Liverpool HIV interactions database, consider addition of oral RPV 25mg once daily (or the patient’s prior ART regimen) during treatment with tecovirimat and for approximately 2 wks after the end of treatment could be considered.* However, some experts believe no additional therapy is necessary during tecovirimat treatment.^†^ Initiation of long-acting cabotegravir/RPV should be avoided during tecovirimat therapy and for 2 wks after conclusion of tecovirimat.^§^
Cidofovir	Tenofovir disoproxil fumarate (TDF)	Nephrotoxicity; probenecid might inhibit excretion of TDF	Coadministration of cidofovir and TDF is not recommended. If concomitant use of TDF and nephrotoxic agents is unavoidable, renal function should be monitored closely. Probenecid might increase serum levels of TDF. Consider use of tenofovir alafenamide (TAF) in place of TDF and monitor for renal adverse events.
	Zidovudine (AZT)	Probenecid increases drug concentration of AZT	Probenecid substantially increases AZT plasma levels, and if coadministered AZT should either be temporarily discontinued or decreased by 50% on the day of cidofovir-probenecid administration to avoid AZT-induced hematological toxicity.
Brincidofovir	Cobicistat (COBI)	Inhibition of OATP1B1, OATP1B3	If concomitant use with brincidofovir is necessary, increase the monitoring for adverse reactions associated with brincidofovir (i.e., elevations in transaminases and bilirubin, diarrhea, or other gastrointestinal adverse events) and postpone the dosing of these antiretrovirals for ≥3 hrs after brincidofovir administration.
	Fostemsavir (FTR)		
	Protease Inhibitors (class)		
	Tenofovir disoproxil fumarate (TDF)	Nephrotoxicity	If concomitant use of TDF and nephrotoxic agents is unavoidable, renal function should be monitored closely.
	Zidovudine (AZT)	Possible reduced renal secretion of AZT	When brincidofovir is coadministered to patients being treated with AZT, they should be closely monitored for AZT-induced hematological toxicity.
Vaccinia immune globulin intravenous	No known or anticipated interactions with antiretroviral therapy	—	—

**Abbreviations:** ART = antiretroviral therapy; CYP = cytochrome P450; NNRTI = non-nucleoside reverse transcriptase inhibitors; OATP = organic anion transporting polypeptide.

* https://hiv-druginteractions.org/checker

^†^ https://cdn.hivguidelines.org/wp-content/uploads/20220715134949/NYSDOH-AI-ARVs-and-Treatments-for-Severe-Monkeypox_7-15-2022_HG.pdf

^§^ https://www.accessdata.fda.gov/drugsatfda_docs/label/2022/212888s005s006lbl.pdf

**Considerations for vaccination:** The safety and immunogenicity of JYNNEOS have been specifically evaluated in persons with HIV infection. Clinical trials demonstrate that JYNNEOS is well-tolerated with similar immunogenicity and rates of adverse events in persons with HIV infection with CD4 cell counts of 200–750 per *μL* and persons without HIV infection (*25*,*26*). In persons with HIV infection with a prior diagnosis of AIDS who were virologically suppressed and had CD4 counts of 100–500 per *μL*, there were no serious safety concerns and the vaccine appeared efficacious based on immunogenicity at standard dosing (*27*). However, immunogenicity among persons with HIV infection who have CD4 cell counts <100 per *μL* or who are not virologically suppressed is not known.

Because ACAM2000 contains a replication-competent, attenuated strain of vaccinia virus, severe localized or systemic complications of ACAM2000 (e.g., progressive vaccinia) can occur in persons with weakened immune systems, including from HIV infection (*15*).

## Interim Guidance

Providers should consider both viral suppression and CD4 count in weighing the risk for severe monkeypox-associated outcomes for any patient with HIV infection. Although severe outcomes have been observed in persons with inadequately treated HIV infection who have CD4 counts ≤350 per *μL* and are likely not virologically suppressed, currently available data are insufficient to define actionable thresholds (*21*,*22*). Until more is known, clinicians should exercise clinical judgement assessing the extent of immunosuppression from HIV and from any other sources, and the relationship of the patient’s immunosuppression to the risk for severe monkeypox illness.

When vaccination is used for prevention of monkeypox in persons with HIV infection, JYNNEOS is preferred over ACAM2000. Based on current recommendations from ACIP, ACAM2000 is contraindicated for persons with HIV infection because of the risk for severe adverse effects resulting from the spread of vaccinia virus (*15*). If high-risk exposures cannot be avoided, immunocompromised persons may receive JYNNEOS in consultation with their health care provider after careful consideration of the risks and benefits (*15*). Clinical efficacy (vaccine effectiveness) of JYNNEOS against monkeypox is unknown, including among persons with HIV infection. Other therapies, including tecovirimat and VIGIV, can be considered for monkeypox postexposure prophylaxis on an individual case-by-case basis, in cases of known high-risk exposure to a confirmed or probable case of infection and clinical conditions that necessitate an alternative option to postexposure vaccination, such as advanced HIV. The efficacy of these therapies as monkeypox postexposure prophylaxis is unknown.

Persons with and without HIV infection should follow the same guidance to protect themselves from monkeypox. Primary prevention of monkeypox includes isolating persons with infection from other persons and their pets, avoiding close contact and sexual activity (including oral, anal, and vaginal sex or sharing of sex toys) with persons with infection, and postexposure vaccination. Persons identified as close contacts of persons with monkeypox should follow any additional guidance from their state or local health department.

## Discussion

Persons with advanced HIV infection or who are not virologically suppressed with ART might be at increased risk for severe disease related to monkeypox. Postexposure prophylaxis and antiviral treatments are available for persons exposed to *Monkeypox virus* or with monkeypox. Vaccination with JYNNEOS is considered safe for persons with HIV infection. Drug interactions between ART and tecovirimat do not preclude coadministration if antiviral therapy for monkeypox is indicated. Prevention and treatment considerations will be updated as more information becomes available.

**Summary**

**What is already known about this topic?**

A multinational monkeypox outbreak disproportionately affecting men who have sex with men, including persons with HIV infection, is ongoing worldwide.

**What is added by this report?**

CDC has developed clinical considerations for prevention and treatment of monkeypox in persons with HIV infection, including pre-exposure and postexposure prophylaxis with JYNNEOS vaccine, treatment with tecovirimat, and infection control.

**What are the implications for public health practice?**

Persons with advanced HIV might be at increased risk for severe monkeypox. Postexposure prophylaxis and antiviral treatments are available for persons with HIV infection. Prompt diagnosis and treatment and enhanced prevention efforts might reduce the risk for severe outcomes.

## Footnotes

†
https://www.washingtonpost.com/health/2022/08/04/monkeypox-public-health-emergency-united-states-becerra/

§
https://www.cdc.gov/poxvirus/monkeypox/transmission.html

¶
https://www.cdc.gov/poxvirus/monkeypox/clinicians/obtaining-tecovirimat.html

**
https://www.cdc.gov/poxvirus/monkeypox/clinicians/smallpox-vaccine.html

††
https://www.cdc.gov/poxvirus/monkeypox/clinicians/monitoring.html

§§
https://www.cdc.gov/poxvirus/monkeypox/considerations-for-monkeypox-vaccination.html

¶¶
https://clinicalinfo.hiv.gov/en/guidelines/hiv-clinical-guidelines-adult-and-adolescent-opportunistic-infections/introduction

***
https://clinicalinfo.hiv.gov/en/guidelines/hiv-clinical-guidelines-adult-and-adolescent-arv/discontinuation-or-interruption

†††
https://www.cdc.gov/poxvirus/monkeypox/clinicians/treatment.html
